# Multidimensional energy poverty and depression among China's older adults

**DOI:** 10.3389/fpubh.2022.977958

**Published:** 2022-09-12

**Authors:** Jianming Hou, Wenjian Zhou, Yang Jiang

**Affiliations:** Northeast Asian Research Center, Jilin University, Changchun, China

**Keywords:** multidimensional energy poverty, depression level, life satisfaction, older adults, China

## Abstract

Older adults often face more pronounced energy inequality in their daily lives, which is one of the reasons for their accumulation of stress or anxiety and may further aggravate their depression. Analyzing the relationship between energy poverty and the depression level of China's older adults will provide policy enlightenment for solving the problems of older adults' relative poverty, energy poverty, and mental poverty and thus promote happy and healthy aging. In this paper, using the data of China Health and Retirement Longitudinal Study in 2018, we used the entropy weighting method to objectively assign weights to 10 indicators reflecting the status of energy poverty and construct a multidimensional energy poverty index for older adults. First, we analyzed the relationship between multidimensional energy poverty and the depression levels of older adults using multiple linear regression model and quantile regression models. Next, we used instrumental variable linear regression model and instrumental variable quantile regression models for endogeneity tests. Then, we performed a robustness check by replacing the core explanatory variable. After that, we conducted heterogeneity analyses by residential area, type of residence, and solitary status. Finally, we analyzed the mediating role of life satisfaction using structural equation modeling. Multidimensional energy poverty has aggravated depression among older adults, and the effect is greater for older adults with higher depression levels. The effect of multidimensional energy poverty on depression is greater for older adults in western China, urban areas, and those who live alone. Multidimensional energy poverty has aggravated depression among older adults by reducing their life satisfaction.

## Introduction

After years of arduous efforts in the battle against poverty, China has made remarkable achievements in poverty alleviation. After achieving moderate prosperity in all respects in 2021, China has entered a stage of poverty mainly characterized by relative poverty. This means that poverty alleviation will change from a tough battle to a prolonged campaign, focusing on solving the problem of relative poverty and thus providing a “Chinese solution” for other developing countries to tackle poverty. According to the seventh national census, the number of older adults aged 60 and above in China was 260 million in 2020, accounting for 18.7% of the total population (1). The scale and proportion of older adults in China have been increasing over the years. China has entered a moderately aging society, and at the same time is facing the problem of “aging before getting rich.” With the change in the direction of poverty alleviation and the acceleration of population aging, the problem of poverty among older adults in China has become increasingly prominent. Compared with other poor populations, the elderly find it more difficult to overcome poverty due to their declining physical functions, single source of income, and declining function of family care. Moreover, they are generally suffering from material, energy, health, and spiritual poverty (2).

As an important dimension of poverty, energy poverty is mainly reflected in the low level, poor structure, and weak capacity of energy use for domestic purposes, resulting in negative impacts on individual health and socioeconomics (3). According to the energy ladder hypothesis, older adults are more likely to suffer from energy poverty after retirement as their income level and socioeconomic status decrease significantly, which further affects their health status (4). Improving energy poverty is an important element of China's sustainable development and the establishment of a social equity system (5). With the deepening understanding of energy poverty in the international community, the United Nations and other international organizations have made the elimination of energy poverty an important development goal.

The United Nations designated 2012 as “the International Year of Sustainable Energy for All” and proposed the “Sustainable Energy for All” initiative, calling for global concerted action to safeguard the right of everyone to have access to modern, clean, and efficient energy for living and to jointly address energy poverty (6). The United Nations Sustainable Development Goal 7 for 2030 is to “ensure access to affordable, reliable, sustainable and modern energy for all” (7), including expanding access to electricity and clean cooking fuels, which is a common task for all countries and everyone. There are still 770 million people worldwide who do not have access to electricity—mostly in developing countries in Africa and Asia. Furthermore, more than 2.5 billion people do not have access to clean cooking fuels, and cooking with traditional biomass, coal, or kerosene causes 2.5 million premature deaths each year, thereby slowing social and economic development and increasing inequality. The outbreak of the coronavirus disease has led to an increase in energy prices, reducing households' ability to purchase energy and reversing the recent progress in areas such as expanding access to electricity and cooking fuels (8). Although China has experienced unprecedented economic growth, it is still a developing country with a very large population, so it is imperative for China to address energy poverty. Access to modern forms of energy is critical to the development, health, education, and other socioeconomic aspects of a country and can help residents improve their living standards and health.

Lewis first introduced the concept of energy poverty, that is, household inability to afford adequate warmth, to illustrate how inadequate energy use affects people's living standards (9). Leach validated the above concept of energy poverty by arguing that low-income households spend a higher proportion of their total household income on energy consumption than middle-income households (10). Boardman proposed the “10% indicator” from the perspective of affordability, arguing that a household can be considered energy poor if its energy consumption expenditure accounts for 10% or more of its total income (11). Many researchers have used this indicator to measure energy poverty (12–14). However, Papada et al. argued that the “10% indicator” is not a good measure of energy poverty because it neglects the fact that some households do not have sufficient access to energy due to their low income (15). Hills proposed the “low-income-high-costs” indicator and defined energy poverty as when residual household income is below the official poverty line while basic energy costs for household living needs are higher than the average (16). This indicator has been used by many researchers (17–19). However, the double relativity of this indicator makes it difficult to isolate the causal relationship in the analysis (20). Nussbaumer et al. (22) first proposed the concept of multidimensional energy poverty and defined it as the inability to obtain clean energy with sustainable human development and social prosperity as the starting point. They constructed a macrolevel multidimensional energy poverty index (MEPI) based on the dual-cutoff approach proposed by Alkire and Foster (21). This index measures energy poverty from several perspectives and has been used by many researchers to measure multidimensional energy poverty in different regions (23–26).

Some scholars have analyzed the health effects of energy poverty. Nawaz et al. found a significant positive impact of energy poverty on the multidimensional health poverty index (consisting of three dimensions—child health, general health, and infectious disease prevalence) of Pakistanis (27). Zhang et al. concluded that energy poverty has an important and significantly negative impact on the average health status of Chinese households (28). Zhang et al. (30) constructed a multidimensional poverty index from five dimensions—cooking, clothing, travel, housing, and education/entertainment and concluded that energy poverty has impaired Chinese residents' physical and mental health (29). Brown et al. measured the health status of Australian residents from different perspectives and found a strong correlation between energy poverty and adverse health status (31). Hailemariam et al. found that energy poverty has increased the possibility of Australian residents suffering physical violence (32). Prakash et al. concluded that energy poverty is positively correlated with obesity among Australian residents (33). Zhang et al. used instrumental variable quantile regression (IVQR) to find a significantly positive impact of energy poverty on depression at the high quantile of depression scores among rural Chinese residents but found no impact at the low and middle quantiles (34). Nie et al. concluded that energy poverty has led to higher depression levels among Chinese adults (35). Lin and Okyere found that energy poverty in Ghana heightenes one's chances of being mentally unhealthy; among the indicators of multidimensional energy poverty, deprivation in household appliance ownership (refrigerator ownership) has the most significant impact on household heads' depression levels (36). From these aforementioned studies, it is clear that there are few studies on the association between multidimensional energy poverty and older adults' mental health, which is the focus of this study.

Other effects of energy poverty have also been analyzed. Getie found that energy deprivation directly or indirectly affects the living standards of individuals in Ethiopia (37). Bukari et al. found that energy poverty significantly increases household health expenditures in Ghana (38). Liu et al. found that energy poverty significantly reduces Chinese residents' subjective wellbeing, with regional, urban–rural, and income heterogeneity. They also found that greater intensity of energy poverty exerts a greater impact on residents' wellbeing (39). Druica et al. believed that energy poverty has a significant impact on the life satisfaction of citizens in many countries (40). From the perspective of different developing countries, Banerjee et al. found that energy poverty reduces the average years of education for citizens (41). Xiao et al. found that energy poverty has a significantly negative impact on individual development through health status (42). Cheng et al. believed that consistent with the disadvantage theory of entrepreneurship, the higher the share of household income spent on energy consumption or energy poverty, the higher the likelihood of becoming an entrepreneur (43). Rafi et al. believed that energy poverty has a significantly negative impact on the human capital development of Indian children (44).

So far, there are few studies on the mechanism by which multidimensional energy poverty affects depression among older adults. Nie et al. concluded that self-rated health and household food expenditure play mediating roles in the impact of energy poverty on depression levels among Chinese adults (35). Some studies have revealed that energy poverty affects residents' welfare and living standards (37–39), and decreases their life satisfaction (40), which increases depression among older adults (45).

Depression is a very common psychological disorder among older adults in China and is affected by a number of social and biological factors. Older adults often face more pronounced energy inequality in their daily lives, which is one of the reasons for their accumulation of stress or anxiety and may further aggravate their depression. This issue needs to be addressed. However, most existing studies have focused on the impact of energy poverty on the health of the population but have paid less attention to the association between energy poverty and the mental health of older adults. Therefore, from the perspective of multidimensional energy poverty, this paper analyzes the relationship between energy poverty and the depression level of China's older adults and explores the heterogeneity across different older adult populations and possible influencing mechanisms. Theoretically, this study improves the existing theoretical system of research on the impact of energy poverty on older adults' mental health. It provides practical policy insights for China to address the problems of relative poverty, energy poverty, and mental poverty of older adults to promote happy and healthy aging.

## Materials and methods

### Data

The data are sourced from the 2018 China Health and Retirement Longitudinal Study (CHARLS)—an interdisciplinary survey project hosted by the National School of Development of Peking University and executed by the Institute of Social Science Survey. The survey aims to collect a set of high-quality microdata of Chinese households and individuals aged 45 years and above to analyze the aging of China's population and promote interdisciplinary research on aging. The survey contains the personal characteristics, health status, energy poverty, and other statuses of older adults, which can satisfy the needs of this study. In this study, older adults aged 60 and above were selected as the study population. After eliminating samples with missing values of variables, the final sample size is 6,222.

### Variables

#### Explained variable

Many studies have shown that the reliability and validity of the CES-D 10 scale are not decreased with the reduction of items (46, 47). Huang et al. found that CES-D 10 scale has high reliability and good discriminant validity, and can effectively measure the level of depression in middle-aged and elderly people in China (48). In the questionnaires of China Health and Retirement Longitudinal Study (CHARLS) and Chinese Longitudinal Healthy Longevity Survey (CLHLS), CES-D 10 is a very important scale to evaluate depression level and has been widely used.

The explained variable is the depression levels among older adults, reflected by the depression scale (CES-D 10), including a total of 10 questions: “I am bothered by small things;” “I have difficulty concentrating when doing things;” “I feel depressed;” “I find it hard to do everything;” “I am hopeful about the future;” “I feel scared;” “I have trouble sleeping;” “I am happy;” “I feel lonely;” and “I don't think I can continue my life.” In this study, the eight questions reflecting negative emotions were assigned values based on the responses as follows: “rarely or not at all” = 0, “not too much” = 1, “sometimes or half of the time” = 2, and “most of the time” = 3. The two questions reflecting positive emotions, that is, “I am hopeful about the future” and “I am happy” were assigned opposite values. The values of the 10 questions were eventually summed to obtain the scores of depression levels, ranging from 0 to 30. A higher depression score indicates a higher depression level and worse mental health status.

#### Core explanatory variable

The core explanatory variable is the MEPI of older adults, and we adopted the framework proposed by Nussbaumer et al. to measure it (22). MEPI under this framework is defined as multiple deprivations of modern energy services for a decent life. we chose ten indicators of energy deprivation embodied in four dimensions: ① access to travel: “own a car;” ② access to entertainment: “own a TV,” “own a computer (tablet);” ③ access to modern housing appliances: “own a refrigerator (freezer),” “own a washing machine,” “own an air conditioner,” “own an air purifier,” “own bathing facilities,” “have heating facilities;” ④ access to clean cooking fuel: “main fuel used for cooking is clean energy.” They were measured on a binary scale, where “yes = 0” or “no = 1.” Compared with the six indicators used in Nussbaumer et al. (22) and the nine indicators used in Zhang et al. (30), we expanded the number of indicators to ten. MEPI was constructed from the above 10 indicators through the Entropy Weight Method, an objective weighting method. A larger MEPI indicates deeper energy poverty experienced by older adults.

Table 1 presents the data statistics and weights for each dimension. Among the samples in this study, 4.74% of the older adults owned a car, 85.71% owned a TV, 10.41% owned a computer (tablet), 75.35% owned a refrigerator (freezer), 67.18% owned a washing machine, 34.96% owned an air conditioner, only 1.67% owned an air purifier, 59.31% owned bathing facilities, 14.56% had heating facilities, and 63.40% of the elderly used clean energy as their main fuel for cooking. We can see that the weight of the variable will increase if the proportion of the older adults who do not own an energy service decreases. “Own an air purifier” had the lowest weight, 0.0024; “Own a TV” had the highest weight, 0.2730.

**Table 1:** Data statistics and weights for each dimension of energy powverty.

**Variables**	**Yes**	**No**	**Weights**
Own a car	295 (4.74%)	5,927 (95.26%)	0.0068
Own a TV	5,333 (85.71%)	889 (14.29%)	0.2730
Own a computer (tablet)	648 (10.41%)	5,574 (89.59%)	0.0154
Own a refrigerator (freezer)	4,688 (75.35%)	1,534 (24.65%)	0.1965
Own a washing machine	4,180 (67.18%)	2,042 (32.82%)	0.1563
Own an air conditioner	2,175 (34.96%)	4,047 (65.04%)	0.0603
Own an air purifier	104 (1.67%)	6,118 (98.33%)	0.0024
Own bathing facilities	3,690 (59.31%)	2,532 (40.69%)	0.1261
Have heating facilities	906 (14.56%)	5,316 (85.44%)	0.0221
Main fuel used for cooking is clean energy	3,945 (63.40%)	2,277 (36.60%)	0.1410

#### Control variables

After reviewing relevant studies and the survey data used (34–36, 49, 50), this study selected the following as control variables: gender, age, marital status, education level, type of residence, work status, physical health, self-rated health, number of household members, availability of health insurance, availability of endowment insurance, and residential area. Educational level was divided into primary school or below, middle school, and high school or above. Work status included unemployed, agricultural work, and non-agricultural work. Physical health status was reflected by activities of daily living scores, ranging from 0 to 18. According to the geographical location and level of economic development, the residential area was divided into western region (including Sichuan, Guizhou, Yunnan, Tibet, Shaanxi, Gansu, Qinghai, Ningxia, Chongqing, Xinjiang, and Guangxi), central region (including Shanxi, Inner Mongolia, Jilin, Heilongjiang, Anhui, Jiangxi, Henan, Hubei, and Hunan), and eastern region (including Beijing, Tianjin, Hebei, Liaoning, Shanghai, Jiangsu, Zhejiang, Fujian, Shandong, Guangdong, and Hainan).

#### Mediator variable

Studies have demonstrated that energy poverty impacts residents' life satisfaction (39, 40), and life satisfaction often has a very important impact on residents' depression. Thus, this study selected life satisfaction as a mediator variable to examine its mediating role between multidimensional energy poverty and older adults' depression. The value range of life satisfaction is 1–5, and the satisfaction decreases as the value increases.

#### Instrumental variable

The existing literature has demonstrated the rationality of community (village) level indicators as instrumental variables for individual indicators (39, 51). Therefore, in this study, the “MEPI at community level,” i.e., the mean of the MEPI of all older adults in the same community, was selected as an instrumental variable. The reason for this is that the multidimensional energy poverty index of the community as a whole is closely correlated with the MEPI of older adults but does not significantly correlate with their other characteristics.

#### Data statistics

Table 2 presents the variables and data statistics. The average depression level of the older adults was 9.750, the mean of MEPI was 0.322, and the average life satisfaction was 2.748, which was between “very satisfied” and “somewhat satisfied.” In terms of control variables, the average age was 66.678 years old, the average activities of daily living were 0.772, the average self-rated health status was 3.209 between “fair” and “poor,” and the average number of household members was 0.849; Among the samples, 54.29% of the elderly were female, 19.72% did not have a spouse, 75.39% had only primary school education or below, 60.27% lived in rural areas, 49.60% were unemployed, 2.86% had no medical insurance, 9.29% had no endowment insurance, and 28.98% of the older adults lived in western region of China.

**Table 2:** Variables and data statistics.

**Continuous variables**	**Mean**	**Standard deviation**	**Min**	**Max**
Depression level	9.750	6.835	0	30
MEPI	0.322	0.261	0	1
Life satisfaction	2.748	0.803	1	5
Age	66.678	6.326	60	95
Activities of daily living	0.772	1.718	0	18
Self-Rated health status	3.209	0.972	1	5
Number of household members	0.849	1.445	0	12
**Categorical variables**	**Categories**	***n***	**Percentages (%)**	**Average depression level**
Gender	Female	3,378	54.29	10.594
	Male	2,844	45.71	8.748
Marital status	No spouse	1,227	19.72	11.015
	With spouse	4,995	80.28	9.439
Education level	Primary school or below	4,691	75.39	10.360
	Middle school	957	15.38	8.439
	High school or above	574	9.23	6.953
Type of residence	Rural	3,750	60.27	10.678
	Urban	2,472	39.73	8.342
Work status	Unemployed	3,086	49.60	9.665
	Agricultural work	2,738	44.01	10.122
	Non-agricultural work	708	11.38	7.881
Medical insurance	No	178	2.86	10.522
	Yes	6,044	97.14	9.727
Endowment insurance	No	578	9.29	10.401
	Yes	5,644	90.71	9.683
Residential area	Western region	1,803	28.98	11.104
	Central region	2,373	38.14	9.808
	Eastern region	2,046	32.88	8.490

As some older adults are engaged in both agricultural and nonagricultural work, the sum of the percentages for all work status is > 100.

### Models

#### Linear regression model

In this paper, we regarded the depression levels among older adults as the explained variable and the MEPI as the core explanatory variable, added various control variables to establish a multivariable linear regression model, in which we used the ordinary least squares (OLS) to analyze the association between multidimensional energy poverty and the depression levels of older adults.


(1) $$Y_{i}=\alpha_{0}+\beta_{00}X_{i}+\sum \beta_{0j}Z_{ij}+\varepsilon_{0i}$$

In Equation (1), *Y*_*i*_ is the *i*-th older adult's depression level; α_0_ is the constant term, and ε_0*i*_ is the error term. *X*_*i*_ is the core explanatory variable, representing the MEPI of *i*-th older adult, respectively, and β_00_ is its coefficient. *Z*_*ij*_ is the *j*-th control variable for the *i*-th older adult, and β_0*j*_ is the coefficient of each control variable.

#### Quantile regression model

As the depression levels of older adults are highly heterogeneous and the same multidimensional energy poverty index may have different impacts on older adults with different depression levels, this paper used QR models to analyze the different relationships between multidimensional energy poverty and the depression levels of older adults at different quantile points to verify whether the findings of the multivariable linear regression model are robust.


(2) $${\text{Q}}_{\text{iq}}\left({\text{Y}}_{\text{i}}\right)=\alpha_{\text{q}}+\beta_{0\text{q}}{\text{X}}_{\text{i}}+\sum \beta_{\text{jq}}{\text{Z}}_{\text{ij}}$$

In Equation (2), *Q*_*iq*_ (*Y*_*i*_) denotes the conditional quantile of depression level given the distribution of core explanatory and control variables, where *q* denotes the quantiles, and 10, 25, 50, 75, and 90% quantiles are selected successively in this paper. The definitions of the remaining variables and parameters are the same as those of the multivariable linear regression model.

#### Instrumental variable linear regression model

Because of the possibility of omitted variables and measurement errors, this study used two-stage least squares (2SLS) to deal with endogeneity in the instrumental variable linear regression model.


(3) $${\text{Y}}_{\text{i}}=\alpha_{1}+\beta_{10}\hat{X_{i}}+\sum \beta_{1j}{\text{Z}}_{\text{ij}}+\varepsilon_{1\text{i}}$$

(4) $${\text{X}}_{\text{i}}\text{=}\gamma_{0}+\delta_{00}{\text{I}}_{\text{i}}+\sum \delta_{0\text{j}}{\text{Z}}_{\text{ij}}{+\text{u}}_{0\text{i}}$$

Equation (4) is the first-stage regression, and *I*_*i*_ is the instrumental variable, i.e., the community-level MEPI. Equation (3) is the second-stage regression, and $\hat{X_{i}}$ is the fitted value of the first-stage regression.

#### IVQR model

As mentioned above, multidimensional energy poverty is endogenous, and the results of the QR models may also be biased. Therefore, in this paper, instrumental variable was added to the QR models, and the IVQR models were used to deal with the endogeneity problem. The specific solution process is based on the study of Chernozhukov and Hansen (52, 53).


(5) $$\text{Y }=\;{\text{X}}^{\prime }\alpha \left(\text{U}\right)+{\text{Z}}^{\prime }\beta \left(\text{U}\right),\text{U}|\text{Z},\text{I}\sim \text{Uniform}\left(0,\text{1}\right)$$

(6) X = f(Z,I,V)

In Equation (5), *Y* represents the depression levels of older adults; *Z* represents the control variables, and *X* represents the endogenous variable. *U* is a random variable that follows a uniform distribution of (0, 1). In Equation (6), *I* is the instrumental variable; *V* is the unobserved disturbance vector, and *V* and *U* are correlated, thus giving rise to endogeneity.

#### Structural equation modeling

In this paper, we used life satisfaction as a mediator variable and developed a SEM to analyze the mechanism through which multidimensional energy poverty impacts the depression level of older adults. The mediating effect model is as follows:


(7) $${\text{Y}}_{\text{i}}=\alpha_{2}+\text{c}{\text{X}}_{\text{i}}+\sum {\text{β}}_{2\text{j}}{\text{Z}}_{\text{ij}}+{\text{ε}}_{2\text{i}}$$

(8) $${\text{M}}_{\text{i}}=\alpha_{3}+\text{a}{\text{X}}_{\text{i}}+\sum \beta_{3\text{j}}{\text{Z}}_{\text{ij}}+{\text{ε}}_{3\text{i}}$$

(9) $${\text{Y}}_{\text{i}}=\alpha_{4}+{\text{c}}^{\prime }{\text{X}}_{\text{i}}+\text{b}{\text{M}}_{\text{i}}+\sum {\text{β}}_{4\text{j}}{\text{Z}}_{\text{ij}}+\varepsilon_{4\text{i}}$$

where *Y*_*i*_, *X*_*i*_, and *M*_*i*_ denote the explained, core explanatory, and mediator variables, respectively. Equation (7) represents the regression equation of the explained variable and the core explanatory variable; Equation (8) represents the regression equation of the mediator variable and the core explanatory variable, and Equation (9) represents the regression equation of the explained variable and both the mediator and core explanatory variables.

## Results

### The relationship between multidimensional energy poverty and depression level

This paper established a multivariable linear regression model as the baseline model to analyze the relationship between multidimensional energy poverty and the depression level of older adults. According to Model 1 in Table 3, the MEPI of older adults is significantly and positively associated with their depression levels at the 1% level; the depression level increases by 2.416 units with each unit increase in the MEPI. We performed a multicollinearity test to eliminate the multicollinearity problems of the multivariable linear regression model.

**Table 3:** Regression results of the relationship between multidimensional energy poverty and depression levels.

**Variables**	**Model 1**	**Model 2**	**Model 3**	**Model 4**	**Model 5**	**Model 6**
	**OLS**	**QR**				
		**Q10**	**Q25**	**Q50**	**Q75**	**Q90**
**Core explanatory variable**						
MEPI	2.416^***^	1.096^***^	2.071^***^	2.452^***^	2.998^***^	3.406^***^
	(0.337)	(0.360)	(0.399)	(0.409)	(0.546)	(0.792)
**Control variables**						
Gender	−1.464^***^	−0.677^***^	−0.933^***^	−1.536^***^	−1.725^**^	−1.529^***^
(Female)	(0.161)	(0.180)	(0.158)	(0.221)	(0.243)	(0.351)
Age	−0.082^***^	−0.038^***^	−0.056^***^	−0.086^***^	−0.111^***^	−0.103^***^
	(0.013)	(0.014)	(0.013)	(0.019)	(0.020)	(0.030)
Marital status	−0.895^***^	−0.319	−0.566^**^	−0.976^***^	−0.907^***^	−1.827^***^
(No spouse)	(0.212)	(0.237)	(0.244)	(0.278)	(0.325)	(0.488)
Middle school	−0.697^***^	0.101	−0.059	−0.620^***^	−1.343^***^	−1.740^***^
(Primary school or below)	(0.205)	(0.210)	(0.231)	(0.214)	(0.309)	(0.453)
High school or above	−1.473^***^	−0.536^**^	−0.914^***^	−1.445^***^	−1.858^***^	−2.807^***^
(Primary school or below)	(0.254)	(0.208)	(0.233)	(0.286)	(0.415)	(0.626)
Type of residence	−0.948^***^	−0.574^***^	−0.883^***^	−1.235^***^	−1.083^***^	−1.092^***^
(Rural)	(0.182)	(0.168)	(0.174)	(0.217)	(0.232)	(0.385)
Agricultural work	0.306^*^	0.164	0.215	0.405^*^	0.210	0.165
(Unemployed)	(0.174)	(0.162)	(0.193)	(0.221)	(0.274)	(0.418)
Non-agricultural work	−0.458^**^	−0.403	−0.182	−0.536^**^	−0.687^*^	−0.344
(Unemployed)	(0.232)	(0.272)	(0.238)	(0.271)	(0.390)	(0.674)
Activities of daily living	0.786^***^	0.566^***^	0.810^***^	0.977^***^	0.963^***^	0.749^***^
	(0.055)	(0.086)	(0.073)	(0.082)	(0.079)	(0.088)
Self-rated health status	1.942^***^	0.928^***^	1.339^***^	2.041^***^	2.501^***^	2.411^***^
	(0.085)	(0.070)	(0.081)	(0.096)	(0.111)	(0.210)
Number of household members	−0.172^***^	−0.079	−0.100^**^	−0.209^***^	−0.178^**^	−0.187
	(0.051)	(0.055)	(0.047)	(0.064)	(0.083)	(0.117)
Medical insurance	0.296	−0.432	0.421	0.268	−0.681	0.637
(No)	(0.459)	(0.372)	(0.441)	(0.699)	(0.717)	(0.758)
Endowment insurance	−0.073	0.030	−0.023	−0.265	−0.239	−0.373
(No)	(0.276)	(0.283)	(0.249)	(0.366)	(0.319)	(0.615)
Central region	−0.864^***^	−0.521^***^	−0.772^***^	−0.897^***^	−1.183^***^	−0.671
(Western region)	(0.194)	(0.188)	(0.228)	(0.269)	(0.297)	(0.447)
Eastern region	−1.630^***^	−1.063^***^	−1.294^***^	−1.632^***^	−2.085^***^	−1.762^***^
(Western region)	(0.198)	(0.216)	(0.207)	(0.264)	(0.323)	(0.448)
Constant	10.452^***^	3.215^***^	5.120^***^	10.052^***^	15.973^***^	19.431^***^
	(1.168)	(1.117)	(1.136)	(1.678)	(1.939)	(2.981)
*R^2^*/pseudo *R^2^*	0.238	0.075	0.103	0.144	0.164	0.152

Robust standard errors in Model 1 are given in parentheses, and the goodness-of-fit statistical measure is R^2^; bootstrap standard errors in Models 2–6 are given in parentheses, with a sample size of 100, and the goodness-of-fit statistical measure is pseudo R^2^;

* ,

** ,

*** represent the significance levels of 10, 5, and 1%, respectively.

As the depression levels among older adults are highly heterogeneous, the same multidimensional energy poverty status may have different impacts on older adults with different depression levels. The QR models were also developed to analyze the association between MEPI and the depression level of older adults in different quantiles. According to Models 2–6 in Table 3, the association between MEPI and the depression level of older adults remains significant at the 1% level with coefficients of 1.096, 2.071, 2.452, 2.998, and 3.406, respectively. The coefficients are greater for older adults with higher depression levels. The results of the multivariable linear regression model and QR models indicate that multidimensional energy poverty aggravates depression among older adults and is detrimental to their mental health.

### Treatment of endogeneity

We performed a Hausman test in the linear regression model, which showed a chi-square value of 8.95, rejecting the original hypothesis at the significance level of 5%, and therefore considered MEPI as an endogenous variable. Obviously, due to the possibility of omitted variables and measurement errors, there was also endogeneity in the quantile regression model. We mentioned above the rationality of community (village) level indicators as instrumental variables for individual indicators (39, 51), so the MEPI at the community level of older adults was selected as an instrumental variable to deal with endogeneity. As a whole, the MEPI at the community level is closely correlated with the individual MEPI of older adults but does not significantly correlate with their other characteristics, which satisfies the correlation between the instrumental variable and the endogenous explanatory variable and the exogeneity between the instrumental variable and the disturbance term. Then we conducted a weak instrumental variable test, and the result showed that the F statistic at the first stage was 1,808.76 (much larger than 10), which excluded the existence of weak instrumental variable. The coefficients of Models 7–12 in Table 4 are 4.213, 2.171, 2.958, 4.120, 5.510, and 6.824, respectively, which are all larger than the corresponding coefficients of Models 1 to 6 in Table 3 and significant at the 1% level. The effect of multidimensional energy poverty on the depression level of older adults becomes significantly larger after treating for endogeneity.

**Table 4:** Estimation results of instrumental variable regression models.

**Variables**	**Model 7**	**Model 8**	**Model 9**	**Model 10**	**Model 11**	**Model 12**
	**2SLS**	**IVQR**				
		**Q10**	**Q25**	**Q50**	**Q75**	**Q90**
MEPI	4.213^***^	2.171^***^	2.958^***^	4.120^***^	5.510^***^	6.824^***^
	(0.683)	(0.735)	(0.654)	(0.682)	(0.914)	(1.228)
Control variables	Yes	Yes	Yes	Yes	Yes	Yes
Constant	10.078^***^	2.234^*^	4.967^***^	9.004^***^	13.834^***^	18.399^***^
	(1.181)	(1.193)	(1.062)	(1.106)	(1.474)	(1.978)

Standard errors in Models 7–12 are given in parentheses;

* ,

*** represent the significance levels of 10 and 1%, respectively.

### Robustness test

Referring to the studies of Zhang et al. (30) and Mendoza et al. (54), when *MEPI* ≥ 0.3, older adults are in a multidimensional energy poverty status; otherwise, they are not in a multidimensional energy poverty status. In this paper, we used multidimensional energy poverty status as the replaced core explanatory variable to conduct a robustness check. According to Table 5, after replacing the core explanatory variable, the coefficients of Models 13–18 are 1.036, 0.516, 0.837, 0.972, 1.161, and 1.378, respectively; after treating for endogeneity, the coefficients of Models 19–24 in Table 6 are 2.459, 1.143, 1.649, 2.399, 3.296, and 4.152, respectively. According to the results of the robustness check, the relationship between multidimensional energy poverty status and the depression level of older adults remains significant.

**Table 5:** Regression results by replacing core explanatory variable.

**Variables**	**Model 13**	**Model 14**	**Model 15**	**Model 16**	**Model 17**	**Model 18**
	**OLS**	**QR**				
		**Q10**	**Q25**	**Q50**	**Q75**	**Q90**
Multidimensional energy poverty status	1.036^***^	0.516^**^	0.837^***^	0.972^***^	1.161^***^	1.378^***^
	(0.170)	(0.212)	(0.206)	(0.210)	(0.239)	(0.343)
Control variables	Yes	Yes	Yes	Yes	Yes	Yes
Constant	10.657^***^	3.386^***^	5.242^***^	10.361^***^	15.985^***^	17.893^***^
	(1.169)	(1.177)	(1.273)	(1.606)	(1.930)	(2.907)
*R^2^*/pseudo *R^2^*	0.236	0.075	0.102	0.142	0.162	0.151

Robust standard errors in Model 13 are given in parentheses, and the goodness-of-fit statistical measure is R^2^; bootstrap standard errors in Models 14–18 are given in parentheses, with a sample size of 100, and the goodness-of-fit statistical measure is pseudo R^2^;

** ,

*** represent the significance levels of 5 and 1%, respectively.

**Table 6:** Estimation results of instrumental variable regression models by replacing the core explanatory variable.

**Variables**	**Model 19**	**Model 20**	**Model 21**	**Model 22**	**Model 23**	**Model 24**
	**2SLS**	**IVQR**				
		**Q10**	**Q25**	**Q50**	**Q75**	**Q90**
Multidimensional energy poverty status	2.459^***^	1.143^***^	1.649^***^	2.399^***^	3.296^***^	4.152^***^
	(0.401)	(0.409)	(0.366)	(0.395)	(0.541)	(0.733)
Control variables	Yes	Yes	Yes	Yes	Yes	Yes
Constant	10.249^***^	2.680^**^	5.301^***^	9.182^***^	13.825^***^	18.256^***^
	(1.184)	(1.198)	(1.068)	(1.103)	(1.455)	(1.951)

Standard errors in Models 19–24 are given in parentheses;

** ,

*** represent the significance levels of 5 and 1%, respectively.

### Heterogeneity analysis

According to the models in Table 1, residential area, type of residence, and the number of household members all have significant correlation with the depression level of older adults. Thus, we used the multivariable linear regression model to analyze heterogeneity by residential area, type of residence, and solitary status (number of household members).

According to Models 25–27 in Table 7, the coefficients of the MEPI are 2.702, 2.290, and 2.317 in the western, central, and eastern regions, respectively, and all are significant at the 1% level. According to Models 28 and 29 in Table 8, the coefficients of MEPI are 1.787 and 3.445 in rural and urban areas, respectively, and both are significant at the 1% level. According to Models 30 and 31 in Table 9, the coefficients of MEPI on the depression level of solitary and non-solitary older adults are 3.224 and 1.297, respectively, and both are significant at the 5% level.

**Table 7:** Estimation results of the multivariable linear regression model by residential area.

**Variables**	**Model 25**	**Model 26**	**Model 27**
	**Western region**	**Central region**	**Eastern region**
MEPI	2.702^***^	2.290^***^	2.317^***^
	(0.610)	(0.537)	(0.615)
Control variables	Yes	Yes	Yes
Constant	8.707^***^	10.986^***^	9.020^***^
	(2.229)	(1.983)	(1.870)
*N*	1,803	2,373	2,046
*R^2^*	0.218	0.229	0.229

Robust standard errors in Models 25–27 are given in parentheses;

*** represents the significance level of 1%.

**Table 8:** Estimation results of multivariable linear regression model by type of residence.

**Variables**	**Model 28**	**Model 29**
	**Rural**	**Urban**
MEPI	1.787^***^	3.445^***^
	(0.421)	(0.567)
Control variables	Yes	Yes
Constant	11.944^***^	8.060^***^
	(1.544)	(1.847)
*N*	3,750	2,472
*R^2^*	0.207	0.245

Robust standard errors in Models 25–27 are given in parentheses;

*** represents the significance level of 1%.

**Table 9:** Estimation results of multivariable linear regression model by solitary status.

**Variables**	**Model 30**	**Model 31**
	**Solitary (number of** **household members** **equal to 0)**	**Non-solitary (number** **of household** **members >0)**
MEPI	3.224^***^	1.297^**^
	(0.433)	(0.539)
Control variables	Yes	Yes
Constant	8.911^***^	13.475^***^
	(1.447)	(2.015)
*N*	3,962	2,260
*R^2^*	0.254	0.222

Robust standard errors in Models 30 and 31 are given in parentheses;

** ,

*** represent the significance levels of 5 and 1%, respectively.

### Mediating effect analysis

In this study, structural equation model was used to analyze the mediating effect of life satisfaction between multidimensional energy poverty and the depression level of older adults. The evaluation criteria of SEM fitting indices are as follows: RMSEA (Root mean squared error of approximation) = 0.000 < 0.05, SRMR (Standardized root mean squared residual) = 0.000 < 0.05, CFI (Comparative fit index) = 1.000 >0.90, and TLI (Tucker-Lewis index) = 1.000 >0.90, which indicates that the model is well-fitted. According to Table 10, the coefficient of MEPI on life satisfaction is 0.161; and the coefficients of life satisfaction and MEPI on depression levels are 2.604 and 1.998, respectively, and both are significant at the 1% level. According to the results in Table 11, the mediating effect of multidimensional energy poverty on the depression level of older adults is 0.418; the direct effect and total effect are 1.998 and 2.416, respectively, and all of them are significant at the 1% level. The mediating effect of life satisfaction accounts for 17.3% of the total effect.

**Table 10:** Results of SEM.

**Variables**	**Life satisfaction**	**Depression level**
Life satisfaction		2.604^***^
		(0.105)
MEPI	0.161^***^	1.998^***^
	(0.045)	(0.322)
Control variables	Yes	Yes
Constant	3.101^***^	2.375^**^
	(0.169)	(1.147)

Bootstrap standard errors are given in parentheses, with a sample size of 500;

** ,

*** represent the significance levels of 5 and 1%, respectively.

**Table 11:** Direct effect, indirect effect, and total effect.

**Effect**	**Coefficient**	**Bootstrap**	**95% confidence**	***P*-value**
		**standard error**	**interval**	
Direct effect	1.998	0.322	[1.367, 2.629]	<0.001^***^
Indirect effect	0.418	0.115	[0.192, 0.644]	<0.001^***^
Total effect	2.416	0.341	[1.748, 3.085]	<0.001^***^

*** represents the significance level of 1%.

## Discussion

In this paper, based on the data of CHARLS in 2018, 10 indicators reflecting the energy poverty status of older adults are synthesized into a MEPI using EWM. First, we analyzed the relationship between multidimensional energy poverty and the depression level of older adults using multiple linear regression and QR models. Next, we used instrumental variable linear regression model and IVQR models for endogeneity treatment. Then, we performed a robustness check by replacing the core explanatory variable. After that, we conducted heterogeneity analyses by residential area, type of residence, and solitary status. Finally, we adopted the SEM to analyze the mediating role of life satisfaction.

Multidimensional energy poverty has aggravated depression among older adults, and similar results have been obtained by other studies (29, 30, 35, 36). After retirement, older adults are removed from their previous positions and have substantially lower incomes and socioeconomic status (55). They then face more pronounced energy inequality in their daily lives, which is one of the reasons for their accumulation of stress or anxiety. As multidimensional energy poverty deepens in older adults, they suffer more pronounced energy inequality and perceive more stress or anxiety. They feel that they are in a lower social class or society is not fair (39), which aggravates their depression. And multidimensional energy poverty will damage physical health, cause poor people to feel low-spirited, lack of happiness, and affect the level of mental health (29). Moreover, multidimensional energy poverty also causes a decline in the quality of life or welfare of older adults, which affects their subjective life satisfaction and may further aggravate their depression. In addition, this effect becomes more significant as depression among older adults increases, which is to some extent the same as the findings of Zhang et al. (34) and Zhang (56). Older adults with severer depression are more fragile and sensitive psychologically and are more easily influenced by the external environment; thus, they are more severely impacted by energy poverty.

Compared with the central and eastern regions, the western region has a low level of socioeconomic development and sparsely populated areas with a significant gap in energy infrastructure development, and the older adults there face higher multidimensional energy poverty. Thus, multidimensional energy poverty has the greatest effect on depression among older adults in the western region, whereas there is little difference in its impact on older adults in the central and western regions, which is inconsistent with the findings of Zhang and Shu (29) and Zhang et al. (30).

The effect of multidimensional energy poverty on depression among urban older adults is greater than that among rural older adults, which is mainly due to the large wealth gap among urban older adults, which is a view shared by several studies (29, 30). Although compared with those in rural areas, urban older adults have lower levels of multidimensional energy poverty, they face greater energy inequality. Urban older adults live in highly modernized and fast-paced cities. Once they have a large gap in energy use with that of others, they experience many inconveniences and relatively large psychological disparities.

The effect of multidimensional energy poverty on depression among solitary older adults is greater than that among non-solitary ones, which is a finding that has not been revealed in existing studies. Compared with solitary older adults, non-solitary older adults have more family members; moreover, their families have higher demand and the willingness and ability to pay for all forms of energy and thus face relatively less energy poverty, which benefits them. Furthermore, family members help older adults to learn to operate various energy-related appliances and devices, and they become more proficient at using them. Due to these two reasons, the effect of multidimensional energy poverty on depression among non-solitary older adults is less significant.

Nie et al. believed that energy poverty affects residents' depression levels through self-rated health and food expenditures (35). In contrast, the present study holds that multidimensional energy poverty decreases older adults' life satisfaction and then increases their depression levels. Energy plays a vital role in the lives of older adults, affecting their access to travel, entertainment, housing appliances, cooking, and other aspects. The elderly are vulnerable to suffer from energy poverty due to inadequate energy infrastructure and family affordability and energy availability constraints, which prevent them from obtaining clean and efficient energy to meet their basic needs. Studies have revealed that energy poverty affects residents' welfare and living standards (37–39), thus decreases their life satisfaction (40), which will increase depression among older adults (45). To sum up, life satisfaction plays a mediating role between multidimensional energy poverty and depression among older adults.

This study has the following limitations. First, this paper analyzed the static relationship between multidimensional energy poverty and the depression level of older adults using cross-sectional data without considering how this association changes over time. Second, energy poverty contains many dimensions and indicators. Second, this paper only synthesized 10 indicators that reflect older adults' energy poverty status to construct a multidimensional poverty index, which may lead to a certain degree of measurement error. Third, multidimensional energy poverty may impact the depression level of older adults through a number of mechanisms, but this paper only considered life satisfaction as a mediator variable.

## Conclusions

The main findings of this study are as follows: ① Multidimensional energy poverty has aggravated depression among older adults, and the effect is greater for older adults with higher depression levels. Older adults with deeper multidimensional energy poverty perceive more stress or anxiety, which aggravates their depression. ② Multidimensional energy poverty has a greater effect on depression among older adults in the western region. The lower level of economic development and relatively backward energy infrastructure in the western region contribute to the deeper multidimensional energy poverty among older adults. ③ The effect of multidimensional energy poverty on depression among urban older adults is more significant. The wide wealth gaps in urban areas cause relatively large psychological disparities among older adults with higher energy poverty. ④ The effect of multidimensional energy poverty on depression among solitary older adults is more significant. Solitary older adults have deeper multidimensional energy poverty and do not have family members to support them in energy use. ⑤ Multidimensional energy poverty aggravates depression among older adults by reducing their life satisfaction.

Based on the above findings, this paper puts forward the following suggestions: ① Investment in energy infrastructure should be increased to ensure that all older adults have access to affordable, reliable, sustainable and modern energy. ② Promote the strategy of coordinated regional development, increase support for the western and other backward regions, make up for shortcomings in energy infrastructure development, and narrow regional disparities. ③ Improve the accessibility of energy services and promote the equalization of energy services in both urban and rural areas. In addition, greater emphasis should be placed on improving the equity of energy accessibility in urban areas. ④ Communities (villages) should increase their attention and livelihood support for older adults living alone, help them reduce multidimensional energy poverty, and guide them to use various energy-related appliances and devices. ⑤ Energy price concessions or quantity concessions should be implemented for older adults to alleviate their energy poverty and thus improve their life quality.

## Data availability statement

Publicly available datasets were analyzed in this study. This data can be found here: China Health and Retirement Longitudinal Study (CHARLS), http://charls.pku.edu.cn/en.

## Ethics statement

The studies involving human participants were reviewed and approved by Ethical Review Committee of Peking University. The patients/participants provided their written informed consent to participate in this study.

## Author contributions

JH and WZ conceived the idea. WZ drafted the manuscript. JH, WZ, and YJ participated in the discussion and put forward valuable opinions. YJ revised and polished this article. All authors have contributed to the article and agree to submit this version.

## Conflict of interest

The authors declare that the research was conducted in the absence of any commercial or financial relationships that could be construed as a potential conflict of interest.

## Publisher's note

All claims expressed in this article are solely those of the authors and do not necessarily represent those of their affiliated organizations, or those of the publisher, the editors and the reviewers. Any product that may be evaluated in this article, or claim that may be made by its manufacturer, is not guaranteed or endorsed by the publisher.

## References

[1] National Bureau of Statistics of China. The 7th Population Census Bulleting. National Bureau of Statistics of China (2021). Available online at: http://www.stats.gov.cn/tjsj/tjgb/rkpcgb/qgrkpcgb/202106/t20210628_1818824.html (accessed April 30, 2022).

[2] Wang CM Shang KJ. Poverty among the elderly and its resolution. Cooperat Econ Sci. (2021) 8:161–3. Available online at: https://kns.cnki.net/kcms/detail/detail.aspx?FileName=HZJJ202108066&DbName=CJFQ2021 (accessed August 22, 2022).

[3] Liao H Tang X Wei YM. Research on energy poverty: current situation and future trend. China Soft Sci. (2015) 8:58–71. Available online at: https://kns.cnki.net/kcms/detail/detail.aspx?FileName=ZGRK201508007&DbName=CJFQ2015 (accessed August 22, 2022).

[4] Fry JM Farrell L Temple JB. Energy poverty and retirement income sources in Australia. Energy Econ. (2022) 106:105793. 10.1016/j.eneco.2021.105793

[5] Li K Liu CF Wei YM. Analysis on China's energy poverty issues. Energy China. (2011) 8:31–35. Available online at: https://kns.cnki.net/kcms/detail/detail.aspx?FileName=ZGLN201108010&DbName=CJFQ2011 (accessed August 22, 2022).

[6] Tang X Liao H Wei YM Zhu BZ. International organizations dealing with energy poverty plan and action. Energy China. (2014) 4:30–4. Available online at: https://kns.cnki.net/kcms/detail/detail.aspx?FileName=ZGLN201404010&DbName=CJFQ2014 (accessed August 22, 2022).

[7] United Nations. The Sustainable Development Goals Report 2021. New York, NY: United Nations Publications (2021).

[8] International Energy Agency. World Energy Outlook 2021. Paris: IEA Publications (2021).

[9] Lewis P. Fuel Poverty Can be Stopped. Bradford: National Right to Fuel Campaign (1982).

[10] Leach G. The energy transition. Energy Policy. (1992) 20:116–23. 10.1016/0301-4215(92)90105-B

[11] Boardman B. Fuel Poverty: From Cold Homes to Affordable Warmth. London: Belhaven Press (1991).

[12] Zhang ZC. Research of rural household energy poverty problems. Energy China. (2014) 1:29–39. Available online at: https://kns.cnki.net/kcms/detail/detail.aspx?FileName=ZGLN201401013&DbName=CJFQ2014 (accessed August 22, 2022).

[13] Heindl P Schuessler R. Dynamic properties of energy affordability measures. Energy Policy. (2015) 86:123–32. 10.1016/j.enpol.2015.06.044

[14] Okushima S. Measuring energy poverty in Japan, 2004-2013. Energy Policy. (2016) 98:557–64. 10.1016/j.enpol.2016.09.005

[15] Papada L Kaliampakos D. A stochastic model for energy poverty analysis. Energy Policy. (2018) 116:153–64. 10.1016/j.enpol.2018.02.004

[16] Hills J. Fuel poverty: The Problem and Its Measurement. London: Interim Report of the Fuel Poverty Review (2011).

[17] Li k Lloyd B Liang XJ Wei YM. Energy poor or fuel poor: what are the differences. Energy Policy. (2014) 68:476–81. 10.1016/j.enpol.2013.11.012

[18] Churchill SA Smyth R Farrell L. Fuel poverty and subjective wellbeing. Energy Econ. (2020) 86:104650. 10.1016/j.eneco.2019.10465028143612

[19] Churchill SA Smyth R. Energy poverty and health: panel data evidence from Australia. Energy Econ. (2021) 97:105219. 10.1016/j.eneco.2021.105219

[20] Li LL Zou XY Wu XY. Overview of the measurement and indicators of energy poverty. Energy Environ Protect. (2020) 34:8–13. Available online at: https://kns.cnki.net/kcms/detail/detail.aspx?FileName=NYBH202006002&DbName=CJFQ2020 (accessed August 22, 2022).6813659

[21] Alkire S Foster J. Counting and multidimensional poverty measurement. J Public Econ. (2011) 95:476–87. 10.1016/j.jpubeco.2010.11.006

[22] Nussbaumer P Bazilian M Modi V. Measuring energy poverty: focusing on what matters. Renew Sust Energy Rev. (2012) 16:231–43. 10.1016/j.rser.2011.07.150

[23] Li K. Approaches to Energy Poverty Assessment and Their Application in China. Beijing: Beijing Institute of Technology Press (2014).

[24] Okushima S. Gauging energy poverty: a multidimensional approach. Energy. (2017) 137:1159–66. 10.1016/j.energy.2017.05.137

[25] Bollino CA Botti F. Energy poverty in Europe: a multidimensional approach. PSL Q Rev. (2017) 70:473–507. Available online at: https://www.webofscience.com/wos/alldb/full-record/WOS:000423909200004 (accessed August 22, 2022).

[26] Sokolowski J Lewandowski P Kielczewska A Bouzarovski S. A multidimensional index to measure energy poverty: the Polish case. Energy Sources Part B Econ Plan Policy. (2020) 15:92–112. 10.1080/15567249.2020.1742817

[27] Nawaz S. Energy poverty, climate shocks, and health deprivations. Energy Econ. (2021) 100:105338. 10.1016/j.eneco.2021.105338

[28] Zhang DY Li JJ Han P. A multidimensional measure of energy poverty in China and its impacts on health: an empirical study based on the China family panel studies. Energy Policy. (2019) 131:72–81. 10.1016/j.enpol.2019.04.037

[29] Zhang ZY Shu HT. Multidimensional energy poverty and resident health. J Shanxi Univer Fin Econ. (2020) 8:16–26. Available online at: https://kns.cnki.net/kcms/detail/detail.aspx?FileName=SXCJ202008003&DbName=DKFX2020 (accessed August 22, 2022)

[30] Zhang ZY Shu HT Yi H Wang XH. Household multidimensional energy poverty and its impacts on physical and mental health. Energy Policy. (2021) 156:112381. 10.1016/j.enpol.2021.112381

[31] Brown H Vera-Toscano E. Energy poverty and its relationship with health: empirical evidence on the dynamics of energy poverty and poor health in Australia. SN Bus Econ. (2021) 1:139. 10.1007/s43546-021-00149-3 34806029 PMC8591752

[32] Hailemariam A Sakutukwa T Yew SL. The impact of energy poverty on physical violence. Energy Econ. (2021) 100:105336. 10.1016/j.eneco.2021.105336

[33] Prakash K Munyanyi ME. Energy poverty and obesity. Energy Econ. (2021) 101:105428. 10.1016/j.eneco.2021.105428

[34] Zhang J He Y Zhang J. Energy poverty and depression in rural China: evidence from the quantile regression approach. Int J Environ Res Public Health. (2022) 19:1006. 10.3390/ijerph19021006 35055829 PMC8776053

[35] Nie P Li QG Sousa-Poza A. Energy poverty and subjective well-being in China: new evidence from the China family panel studies. Energy Economics. (2021) 103:105548. 10.1016/j.eneco.2021.105548

[36] Lin BQ Okyere MA. Multidimensional energy poverty and mental health: micro-level evidence from Ghana. Int J Environ Res Public Health. (2020) 17:6726. 10.3390/ijerph17186726 32942745 PMC7558081

[37] Getie EM. Poverty of energy and its impact on living standards in Ethiopia. J Electr Comp Eng. (2020) 2020:7502583. 10.1155/2020/7502583

[38] Bukari C Broermann S Okai D. Energy poverty and health expenditure: evidence from Ghana. Energy Econ. (2021) 103:105565. 10.1016/j.eneco.2021.10556512342139

[39] Liu ZM Deng MY Cui ZW Cao H. Impact of energy poverty on the welfare of residents and its mechanism: an analysis based on CGSS data. China Soft Sci. (2020) 8:143–63. Available online at: https://kns.cnki.net/kcms/detail/detail.aspx?FileName=ZGRK202008014&DbName=CJFQ2020 (accessed August 22, 2022).

[40] Druica E Goschin Z Ianole-Calin R. Energy poverty and life satisfaction: structural mechanisms and their implications. Energies. (2019) 12:3988. 10.3390/en12203988

[41] Banerjee R Mishra V Maruta AA. Energy poverty, health and education outcomes: evidence from the developing world. Energy Econ. (2021) 101:105447. 10.1016/j.eneco.2021.105447

[42] Xiao YM Wu H Wang GH Wang SR. The relationship between energy poverty and individual development: exploring the serial mediating effects of learning behavior and health condition. Int J Environ Res Public Health. (2021) 18:8888. 10.3390/ijerph18168888 34444636 PMC8393606

[43] Cheng ZM Tani M Wang HN. Energy poverty and entrepreneurship. Energy Econ. (2021) 102:105469. 10.1016/j.eneco.2021.105469

[44] Rafi M Naseef M Prasad S. Multidimensional energy poverty and human capital development: empirical evidence from India. Energy Econ. (2021) 101:105427. 10.1016/j.eneco.2021.105427

[45] Park JH Min S Eoh Y Park SH. The elderly living in single-person households in south Korea: a latent profile analysis of self-esteem, life satisfaction, and depression. Qual Life Res. (2021) 30:1083–92. 10.1007/s11136-020-02693-1 33175308

[46] Lee AEY Chokkanathan S. Factor structure of the 10-item CES-D scale among community dwelling older adults in Singapore. Int J Geriatr Psychiatry. (2008) 23:592–7. 10.1002/gps.1944 18023070

[47] Bjorgvinsson T Kertz SJ Bigda-Peyton JS McCoy KL Aderka IM. Psychometric properties of the CES-D-10 in a psychiatric sample. Assessment. (2013) 20:429–36. 10.1177/1073191113481998 23513010

[48] Huang QB Wang XH Chen G. Reliability and validity of 10-item CES-D among middle aged and older adults in China. China J Health Psychol. (2015) 7:1036–41. Available online at: https://kns.cnki.net/kcms/detail/detail.aspx?FileName=JKXL201507024&DbName=CJFQ2015 (accessed August 22, 2022).

[49] Hu Y Li B. Temporal trend of prevalence of depressive symptoms and associated factors among Chinese older adults: an analysis based on the CHARLS panel data. Chin Gen Pract. (2021) 24:3281–7. Available online at: https://kns.cnki.net/kcms/detail/detail.aspx?FileName=QKYX202126004&DbName=CJFQ2021 (accessed August 22, 2022).35869767

[50] Wu NW Yang F Xia J Ma TP Yu C Li NX. Analysis of the status of depression and the influencing factors in middle-aged and older adults in China. J Sichuan Univer. (2021) 5:767–71. Available online at: https://kns.cnki.net/kcms/detail/detail.aspx?FileName=HXYK202105010&DbName=CJFQ2021 (accessed August 22, 2022). 34622590 10.12182/20210960507 PMC10408879

[51] Qi LS. A study on the anti-poverty, income increasing and re-distributional effects of new cooperative medical system. J Quant Techn Econ. (2011) 8:35–52. Available online at: https://kns.cnki.net/kcms/detail/detail.aspx?FileName=SLJY201108004&DbName=CJFQ2011 (accessed August 22, 2022).

[52] Chernozhukov V Hansen C. An IV model of quantile treatment effects. Econometrica. (2005) 1:245–61. 10.1111/j.1468-0262.2005.00570.x

[53] Chernozhukov V Hansen C. Instrumental quantile regression inference for structural and treatment effect models. J Econom. (2006) 2:491–525. 10.1016/j.jeconom.2005.02.009

[54] Mendoza CB Cayonte DDD Leabres MS Manaligod LRA. Understanding multidimensional energy poverty in the Philippines. Energy Policy. (2019) 133:110886. 10.1016/j.enpol.2019.110886

[55] Zhou WJ Hou JM Sun M Wang C. The impact of family socioeconomic status on elderly health in China: based on the frailty index. Int J Environ Res Public Health. (2022) 19:968. 10.3390/ijerph19020968 35055790 PMC8775784

[56] Zhang J. The bidirectional relationship between body weight and depression across gender: a simultaneous equation approach. Int J Environ Res Public Health. (2021) 18:7673. 10.3390/ijerph18147673 34300124 PMC8304982

